# Parkinson’s disease case ascertainment in a large prospective cohort

**DOI:** 10.1371/journal.pone.0251852

**Published:** 2021-05-19

**Authors:** Srishti Shrestha, Christine G. Parks, Marie Richards-Barber, Honglei Chen, Dale P. Sandler

**Affiliations:** 1 Epidemiology Branch, National Institute of Environmental Health Sciences, Research Triangle Park, Durham, North Carolina, United States of America; 2 Westat, Inc., Durham, North Carolina, United States of America; 3 Department of Epidemiology and Biostatistics, College of Human Medicine, Michigan State University, East Lansing, Michigan, United States of America; Istituto Di Ricerche Farmacologiche Mario Negri, ITALY

## Abstract

**Background:**

In epidemiologic studies where physician-based case adjudication is not feasible, Parkinson’s disease (PD) case ascertainment is often limited to self-reports which may not be accurate. We evaluated strategies to identify PD cases in the Agricultural Health Study (AHS).

**Methods:**

Doctor-diagnosed PD was self-reported on all cohort-wide surveys; potential cases were also identified from death certificates. Follow-up surveys asked about PD-related motor and non-motor symptoms. For PD confirmation, we collected additional diagnosis, symptom, and treatment data from 510 potential PD cases or their proxy (65% of those contacted) in a supplemental screener and obtained medical records for a subset (n = 65). We classified PD cases using established criteria and screener data.

**Results:**

Of 510 potential PD cases, 75% were considered “probable” or “possible”; this proportion increased among participants diagnosed by a specialist (81.2%), taking PD medication (85.2%), or reporting ≥5 motor symptoms (86.8%) in a regular AHS survey. Of those with medical records, 93% (57 of 61) of probable or possible PD was confirmed. Never-smoking and non-motor and motor symptoms reported in prior AHS surveys were more common with probable/possible PD than unconfirmed PD.

**Conclusion:**

In this retrospective PD case ascertainment effort, we found that PD self-report with information on motor symptoms or medications may be a reasonable alternative for identifying PD cases when physician exam is not feasible. Because of intervening mortality, screeners could not be obtained from about one-third of those contacted. Thus, findings warrant replication.

## Introduction

Parkinson’s disease (PD) is a chronic progressive neurodegenerative disorder, predominantly affecting older adults [1]. Clinicians rely on the presence of cardinal motor signs including bradykinesia, tremor, rigidity, and postural instability to diagnose PD, though establishing a clinical diagnosis of PD can be challenging and typically requires thorough clinical evaluations of neurological signs and symptoms and response to dopaminergic treatment.

PD etiology is largely unknown. Well-designed epidemiological studies have the potential to provide information on relevant risk factors. Retrospective case-control studies (where disease diagnosis predates exposure measurement), though efficient, are prone to biases associated with recall or reverse causality. In contrast, large prospective cohort studies with long-term follow up and with collection of exposure data prior to diagnosis are less subject to reverse causation, although it is possible that, for conditions such as PD with a long prodromal period, individuals with suspect diagnoses could still preferentially report prior exposures. However, systematic PD ascertainment (for example, with cohort-wide clinical screening or exams) is often infeasible due to practical constraints and cost. Although secondary data sources such as medication inventories, medical records, and administrative data, have been used for PD ascertainment in epidemiological studies [2–8], in large population-based cohorts, researchers often must rely on self-reported physician-made diagnosis, with or without additional clinical information. Few studies have evaluated strategies to optimize self-report for PD case ascertainment in large cohorts [6,9–11], and none specifically in the context of rural farming populations.

The Agricultural Health Study (AHS) is an ongoing prospective cohort study of over 80,000 participants (pesticide applicators and spouses enrolled in 1993–1997), with follow-up surveys updating health status every 5–6 years [12]. Due to its long-term follow-up and large sample of aging participants, along with the collection of detailed prospective data on agricultural factors, the AHS is well-suited to study environmental risk factors for PD. A previous AHS publication analyzed PD cases based on self-report of physician diagnosis [13]. In a case-control study nested within the cohort, self-reported prevalent and incident PD cases identified through the first follow-up were further clinically examined by movement disorder specialists [14], and self-reports were shown to have relatively high accuracy. With additional follow-ups, we have accrued potential cases through self-report and death certificates, and recently attempted to confirm potential PD cases by collecting and evaluating additional information on symptoms and treatment from cases or from proxy respondents for cases who were deceased or too ill. We also sought medical records from treating physicians for a subset of participants.

Here, we investigate the validity of case ascertainment using self-report of doctor-diagnosed PD in AHS cohort-wide surveys, compared with adjudication based on established diagnostic criteria [15] after reviewing detailed information on symptoms and treatment collected in the recent PD confirmation effort, or with medical records. We extend this comparison by stratifying on self-reported information related to PD diagnosis (i.e., diagnosis by a specialist, medication use, and PD-related motor symptoms) collected on cohort-wide surveys to determine their utility for improving PD case identification, and exploring the strength of associations between PD at varying levels of diagnostic certainty and factors known to be associated with PD.

## Materials and methods

### Study population

In 1993–1997 (Phase 1), 52,394 private pesticide applicators (97.4% male, mainly farmers) from North Carolina and Iowa enrolled in the AHS [12]. Spouses of married applicators [n = 32,345 (75% response); 99.3% female] also enrolled. Participants (n = 84,739) were followed through three subsequent surveys: Phase 2, completed by 33,456 farmers and 23,796 spouses in 1999–2003; Phase 3, completed by 24,170 farmers and 19,959 spouses in 2005–2010; and Phase 4, completed by 24,145 farmers and 18,186 spouses in 2013–2016 (S1 Fig). A shorter Phase 4 survey could be completed by proxies for participants who were incapacitated or deceased; this less detailed survey was completed for 2,549 of the 24,145 applicators and 1,001 of the 18,186 spouses. At the time participants were enrolled into the study, written informed consent was not required, and returning the questionnaires amounted to consent. A cover letter included with questionnaire provided information on the study, its voluntary nature, and costs/benefits and risks associated with the study participation, and all participants implied informed consent by returning study questionnaires. Likewise, during computer assisted follow-up interviews, participants were provided the information they needed to decide to continue to participate in the study by completing follow up, and their verbal agreement to proceed with the interview allowed interviewers to continue. Institutional review boards at the National Institute of Environmental Health Sciences and the National Cancer Institute approved the study protocol.

### PD case ascertainment

Participants were asked about doctor-diagnosed PD in all AHS surveys. At enrollment, participants were asked, “has a doctor ever told you that you had Parkinson’s disease?” Follow-up surveys collected more information: age at diagnosis; if a diagnosis was confirmed by a neurologist/movement disorder specialist; if they had ever taken medications for PD; and if their symptoms improved after medications. For the current analysis, participants providing a positive response to the question on doctor-diagnosed PD (regardless of their response to other questions) were considered self-reported PD.

Potential PD cases were also identified via linkage to the National Death Index and state death registries through December 31, 2016, with International Classification of Disease, 9^th^ revision (ICD-9) code 332.0 as the underlying or contributing cause of death.

### Ancillary studies to confirm PD diagnosis

#### Previous effort to compare self-reports with clinical examinations in 2002–2008

The Farming and Movement Evaluation (FAME) study was a nested case-control study within the AHS, conducted in 2002–2008 (S1 Fig) [14]. In this study, self-reported PD cases and selected controls were clinically evaluated. Briefly, the study targeted the 170 participants who self-reported PD in Phase 1 or 2 surveys and 644 participants without PD. Of those, 137 potential cases (including four controls who self-reported PD before the in-home FAME exam) and 383 controls were eligible and agreed to participate. They underwent in-person evaluations including a standardized medical and neurological history and examination (more detail elsewhere [14]). Movement disorder specialists evaluated all potential cases and 5% of controls; neurologist-trained technicians evaluated the remaining controls. Two movement disorder specialists independently reviewed all diagnostic information including medical records and in-person evaluations to confirm PD diagnosis for all potential PD cases and for controls when PD was suspected by the clinical examiner. Of the 137, 115 (84%) were confirmed as having a diagnosis of PD. Of the 383 controls, none were considered to have PD.

#### Updated PD confirmation effort

**Fig 1:**
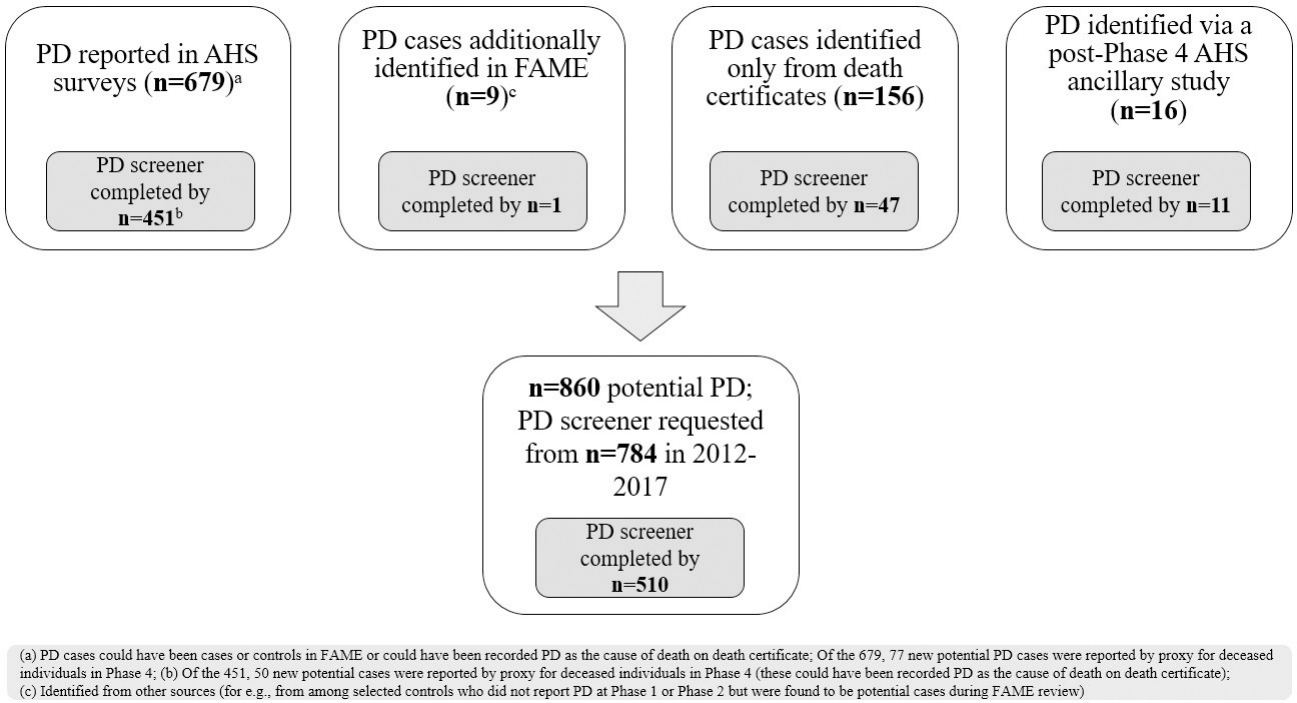
Potential Parkinson’s Disease (PD) cases in the Agricultural Health Study, Iowa and North Carolina, 1993–2016.

Between 2012 and 2017, we attempted to confirm the diagnosis of 860 PD cases identified through Phase 4 on AHS surveys or death certificates (ICD-9 code 332.0 (Figs 1 and S1). We contacted participants with potential PD, or their proxy if patients were deceased or ill, by telephone or mail to complete a screening questionnaire (referred to as “PD screener” here onwards). The screener included a detailed history of PD symptoms, a parkinsonism screening questionnaire [16], PD treatments, and potential alternative diagnoses (S1 Document). We requested for medical record release (from either a treating or diagnosing physician, or neurologist/movement disorder specialist seen within the past five years). We excluded 76 self-reported cases including those deceased before 2006 or identified after PD confirmation study closure, and some prevalent cases reported at enrollment, leaving 784 potential PD cases eligible for the current study; of these, 65% (n = 510) completed the screener. Those without a completed screener were more likely to be deceased, older, spouses/females, and from North Carolina. Of those without completed screener, 93% were deceased at the time of this PD confirmation effort (compared to 36% of the 510 with a screener), with 47% already deceased before 2010 (note: screeners were sought from a proxy even though participants were deceased). Further, death certificate was the only source of PD identification for 38% of those without a screener.

We classified cases as “probable” and “possible” PD, using an algorithm based on the Gelb PD diagnostic criteria (referred to as “screener-based algorithm” here onwards, S1 Table), which we applied to the PD screener responses [15]. Those who did not meet the criteria were classified as “questionable”, “other neurological conditions”, or “not PD”. Those who had some PD-related information, but not enough information to meet the criteria for “probable” or “possible PD,” were considered “questionable.” The screener data were evaluated blind to prior PD status in FAME or information on treatment and symptoms provided in prior cohort-wide surveys.

Of the 510 potential PD cases with completed screener, only 116 (23%) consented to release their medical record. Of those who consented, we obtained medical records or forms confirming diagnosis by physicians for 65 participants prior to study closure (56% of those who consented; 13% of those screened) which were reviewed, blinded to the algorithm diagnosis.

#### Additional cohort-wide data collection on motor and non-motor symptoms

In addition to PD diagnosis, Phase 3 and 4 surveys collected data on an abbreviated list of PD symptoms (S3 Table). In Phase 3, participants were asked seven questions on PD motor symptoms during the last 12 months, and in Phase 4, they were asked about having ever had six PD motor symptoms. In Phase 4, participants were also asked about non-motor symptoms commonly experienced by PD patients including impaired olfaction and dream-enacting behavior [17].

### Statistical analysis

We considered potential PD cases who met the criteria for “probable” or “possible” as “screener-confirmed PD”, those who did not as “unconfirmed PD”, and the rest of the cohort with no evidence of PD as “non-cases”. We evaluated how well self-reported PD in AHS surveys overall compared with screener-confirmed case status. We were interested in determining utility of adding self-reported information related to PD diagnosis (i.e., diagnosed by a movement disorder specialist/neurologist, currently taking medications, and presence of motor symptoms) for identifying clinical PD cases. Since the Phase 4 survey was closest in time to the PD confirmation effort, we examined how using this information along with self-report for cases reported in Phase 4 compared with classification based on the screener data. We also compared cases classified by our screener-based algorithm with cases confirmed by medical records among those with available data.

As PD prevalence is higher in older individuals, men, and non-smokers, we evaluated these characteristics in relation to screener-based case classification. We used polytomous logistic regression models with “screener-confirmed PD”, “unconfirmed PD”, and “non-cases” as categories of the dependent variable to evaluate baseline variables, namely age, sex, state of residence, education, and smoking status, as potential predictors, using data for the entire cohort (n = 84,389); we report odds ratios (OR) and 95% confidence intervals (CI). We used missing indicators to denote missing data for education and smoking status to retain all potential PD cases in the analysis. We also examined whether individual non-motor and motor symptoms (yes versus no) and number of motor symptoms (categorized as 0–2, 3–4, and ≥ 5) reported in Phase 3 (n = 43,969) and in Phase 4 (n = 42,193) were differentially associated with “screener-confirmed PD” as compared to “unconfirmed PD” using polytomous logistic regression, adjusting for baseline covariates; these analyses were limited to Phase 3 and Phase 4 participants respectively, resulting in smaller analytical sample sizes.

## Results

### PD screeners

Of the 510 participants with potential PD or their proxies who completed the screener, the screener-based algorithm classified 75% as “probable” (32.5%) or “possible” PD (42.5%); 4% were deemed “questionable”, 9% “other neurological conditions”, and 12% “no PD” (Table 1). Other neurological conditions were dementia, Alzheimer’s disease, dementia with Lewy bodies, frontotemporal dementia, multiple system atrophy, progressive supranuclear palsy, Guillain-Barré syndrome, stroke, and essential tremor; all were identified based on updated diagnoses provided by participants at the time of completion of the screener. Proxy respondents completed the screener for 49% of participants. Deceased participants with proxies also tended to be classified as “possible” rather than “probable” due to missing information on screeners.

**Table 1:** Among participants (or their proxies) who completed the Parkinson’s Disease (PD) screener (N = 510), classification of potential PD cases applying criteria analogous to the Gelb diagnostic criteria using self- or proxy- reported neurological symptoms and PD treatments reported at the screener; full screener sample and subsets reporting PD in the Agricultural Health Study Surveys, Iowa and North Carolina, 1993–2016.

PD Classification^a^			PD screener respondent						AHS follow-up survey report of PD			
	All potential PD^b^		Self^c^		Proxy ill^d^		Proxy deceased^e^		Any report^f^		Self-report only^g^	
	(n = 510)		(n = 260)		(n = 64)		(n = 186)		(n = 451)		(n = 401)	
	n	%	N	%	n	%	n	%	n	%	n	%
Probable	166	32.5	94	36.2	23	35.9	49	26.3	158	35	151	37.9
Possible	217	42.5	104	40	26	40.6	87	46.8	192	42.6	163	40.7
Questionable	21	4.1	10	3.8	5	7.8	6	3.2	18	4	16	4
Other neurological condition	45	8.8	21	8.1	8	12.5	16	8.6	34	7.5	31	7.7
No PD	61	12	31	11.9	2	3.1	28	15.1	49	10.9	39	9.7

^a^ Self-reported (or proxy-reported when participants were deceased or ill) information on the screener was evaluated using the criteria analogous to the established diagnostic criteria to classify potential PD into “probable”, “possible”, “questionable”, “other neurological condition”, and “no PD”.

^b^510 includes potential PD identified from all sources.

^c^Screener completed by participants.

^d^Proxy respondent for ill participants.

^e^Proxy responded for deceased participants.

^f^Includes only those whose PD were identified from any AHS survey (Of the 451, 50 new potential cases were reported by proxy for deceased individuals in Phase 4).

^g^Analysis was conducted excluding 50 new potential cases reported by proxy for deceased individuals in Phase 4.

Among those whose PD was identified from any of the AHS surveys (n = 451, Table 1), the screener-based algorithm classified 77.6% as either “probable” (35%) or “possible” (42.6%). In the analysis restricted to newly reported potential PD cases in Phase 4 survey (n = 266), 74.8% were “probable” (26.3%) or “possible PD” (48.5%) (Table 2). Participants whose PD was reported as being diagnosed by a movement disorder specialist or neurologist in the cohort-wide Phase 4 survey were more likely to be classified (based on the screener) as “probable PD” (81.4%) and less likely to be classified as “no PD” (5%) compared to those who reported not being diagnosed by a specialist (63.6% and 18.2%, respectively); a similar pattern was observed for those who reported currently taking medications versus those who did not. Further, participants reporting fewer motor symptoms (0–2) in the Phase 4 survey were more likely to be classified as “no PD” and less likely to be “probable” or “possible PD”, compared to those reporting ≥ 5 motor symptoms. Sixty-two clinically confirmed PD cases in FAME also completed the screener; of these, 92% were classified as either “probable” (60%) or “possible” PD cases (32%) (S2 Table).

**Table 2:** Among those who newly reported Parkinson’s Disease (PD) in AHS Phase 4 and who completed the screener (N = 266), classification of potential PD cases applying criteria analogous to the Gelb Diagnostic Criteria using self- or proxy- reported neurological symptoms and PD treatment reported at the screener, Iowa and North Carolina, 1993–2016.

	Overall		Confirmed by a specialist^b^				PD medication^b^				Number of motor symptoms ((n (%))^b^					
	(n = 266)		No (n = 22)		Yes (n = 221)		No (n = 36)		Yes (n = 203)		0 to 2 (n = 48)		3 to 4 (n = 73)		5 to 6 (n = 76)	
PD Classification^a^	n	%	n	%	n	%	n	%	n	%	n	%	n	%	n	%
Probable	70	26.3	0	0	69	31.2	2	5.6	66	32.5	9	18.8	16	21.9	32	42.1
Possible	129	48.5	14	63.6	111	50.2	17	47.2	107	52.7	16	33.3	43	58.9	34	44.7
Questionable	15	5.6	2	9.1	13	5.9	4	11.1	10	4.9	3	6.3	7	9.6	3	3.9
Other	19	7.1	2	9.1	17	7.7	5	13.9	14	6.9	1	2.1	7	9.6	5	6.6
No PD	33	12.4	4	18.2	11	5	8	22.2	6	3	19	39.6	0	0	2	2.6

^a^Self-reported (or proxy-reported when participants were deceased or ill) information on the screener was evaluated using the criteria analogous to the established diagnostic criteria to classify potential PD into “probable”, “possible”, “questionable”, “other neurological condition”, and “no PD”.

^b^Classification was stratified by three variables reported in AHS Phase 4: If PD confirmed by a specialist; if participants were taking PD medications; and number of motor symptoms they ever exhibited.

### PD screeners and medical record review

Of the 65 participants whose medical records or physicians’ diagnosis confirmation forms were obtained, 61 were considered “probable” or “possible” cases by PD screener and four “unconfirmed” cases (Table 3). Of these, 59 (90.8% overall) were confirmed PD by medical record review, including 57 of the 61 (93.4%) screener-defined “probable” or “possible” PD cases and two of the four “unconfirmed cases”.

**Table 3:** Percent of Parkinson’s Disease (PD) cases confirmed by medical record or physician by Gelb criteria^a^ classification (n = 65) in the Agricultural Health Study, Iowa and North Carolina, 1993–2016.

PD-confirmed by medical records	Probable		Possible		Questionable		Other neurological condition	
	n	%	n	%	n	%	n	%
Unclear	1	2.9	3	11.1	0	0	2	66.7
Yes	33	97.1	24	88.9	1	100	1	33.3

^a^ Self-reported information on the screener was evaluated using criteria analogous to the established diagnostic criteria to classify potential PD into “probable”, “possible”, “questionable”, “other neurological condition”, and “no PD”

### Factors associated with PD identification and confirmation

Table 4 presents participants’ enrollment characteristics. Potential PD cases initially identified from AHS self-reports and death certificate (n = 860) were more often older, applicators, and men, tended to have lower education, and were less likely to be current smokers compared to the rest of cohort. Characteristics of screener-confirmed PD (i.e., “probable” or “possible” (n = 383) and screener “unconfirmed” cases (n = 127)) were much more similar to each other than to the rest of the cohort (n = 83,879). The association with male sex was stronger for screener confirmed (OR = 2.7) than for unconfirmed (OR = 1.6) cases though confidence intervals overlap. Similarly, the inverse association with current smoking was more pronounced for confirmed cases (OR = 0.3; 95% CI: 0.2, 0.6) than for unconfirmed cases (OR = 0.7; 95% CI: 0.4, 1.4). The percentage of screener-confirmed self-reported PD was lower among those aged > 65 years, spouses/females, from North Carolina, and current smokers.

**Table 4:** Enrollment Characteristics of potential cases and non-cases and comparison of confirmed and unconfirmed cases with noncases in the agricultural health study (N = 84,739), Iowa and North Carolina. 1993–2016.

	Non cases n = 83,879	Potential PD^a^ n = 860	PD defined using screener-based algorithm (n = 510)						% Confirmed^d^
			Confirmed PD^b^ (n = 383)			Unconfirmed PD^b^ (n = 127)			
	%	%	%	OR ^c^	95% CI	%	OR^c^	95% CI	
Age at enrollment									
≤45 years	49.3	8.8	11	1.0		11.8	1.0		73.7%
46–55 years	23.8	19.2	23.2	4.9	3.4, 7.1	22.0	4.2	2.2, 7.9	76.1%
56–65 years	18.3	39.3	44.1	11.8	8.3, 16.7	35.4	9.0	5.0, 16.4	79.0%
>65 years	8.7	32.7	21.7	11.7	8.0, 17.2	30.7	16.5	8.9, 30.5	68.0%
Participant									
Spouse	38.3	24.7	20.6			29.1			68.1%
Applicator	61.7	75.3	79.4			70.9			77.1%
Sex									
Female	39.7	24.7	20.1	1.0		29.1	1.0		68.0%
Male	60.3	75.3	79.9	2.7	2.1, 3.5	70.9	1.6	1.1, 2.4	77.3%
State									
Iowa	63.3	61.6	65.8	1.0		59.8	1.0		76.8%
North Carolina	36.7	38.4	34.2	0.7	0.6, 0.9	40.2	0.9	0.6, 1.3	72.0%
Education									
≤High school	49.7	63	64	1.0		56.7	1.0		77.3%
1–3y beyond high school	24.4	18.8	17.8	0.9	0.7, 1.2	22.8	1.3	0.9, 2.1	70.1%
≥ College graduate	18.1	13.3	14.9	1.0	0.7, 1.3	15.7	1.2	0.7, 2.0	74.0%
Missing	7.9	4.9	3.4	0.5	0.3, 1.0	4.7	0.8	0.3, 1.9	68.4%
Smoking status									
Never smoker	58.9	60.7	63.4	1.0		59.1	1.0		76.4%
Former smoker	25.3	31.7	30.5	0.7	0.5, 0.9	31.5	0.8	0.5, 1.2	74.5%
Current smoker	13.6	5.8	4.7	0.3	0.2, 0.6	8.7	0.7	0.4, 1.4	62.1%
Missing	2.3	1.7	1.3	0.5	0.2, 1.2	0.8	0.3	0.0, 1.9	83.3%

^a^Includes all potential PD cases identified from all data sources.

^b^We categorized potential PD cases that met the criteria for “probable” or “possible” PD (when PD screener data were evaluated) as “screener-confirmed PD” and those that did not as “unconfirmed PD”, and the rest of the cohort with no evidence of PD as “non-cases”.

^c^Odds ratios (OR) obtained from polytomous logistic regression with “screener-confirmed”, “unconfirmed” and “non-cases” modeled as a dependent variable; ORs are mutually adjusted for other covariates and age modeled as a continuous covariate for mutual adjustment for other covariates.

^d^Percentage of potential PD confirmed by the screener-based algorithm–by participants’ enrollment characteristics.

We also examined self-reported motor and non-motor symptoms at Phases 3 and 4 in relation to PD screener-defined case status (S3 Table). Compared to the 5% of non-cases reporting ≥ 3 motor symptoms in Phase 3 or 4, 54% of the screener-confirmed cases reported ≥ 3 symptoms at Phase 3 and 84% at Phase 4. The corresponding percentages for unconfirmed cases were 32% at Phase 3 and 50% at Phase 4. Adjusting for baseline covariates and compared to non-cases, these symptoms, individually and collectively, were more strongly associated with screener-confirmed PD than with unconfirmed. For example, ORs for associations of having ≥ 5 symptoms in Phase 3 with “screener-confirmed PD” and “unconfirmed PD” were 52.2 (95% CI: 38.2, 71.4) and 14.2 (95% CI: 7.8, 25.7) respectively; and analogous ORs for Phase 4 symptoms were 449.8 (95% CI: 293, 690.6) and 28.9 (95% CI: 14.6, 57.3). Similarly, at Phase 4, both poor olfaction and dream-enacting behavior were more strongly associated with screener-confirmed (corresponding ORs = 10.9 (95% CI: 8.3, 14.3), and 8.5 (95% CI: 6.4, 11.2)) than unconfirmed (3.3 (95% CI: 2.0, 5.6), and 2.0 (95% CI: 1.0, 4.0)).

### Classification of deceased participants

Of the 860 participants with potential PD, 501 were deceased by the end of this case confirmation effort (December 31, 2016). Of the 501 potential PD cases who were deceased, 345 had reported physician-diagnosed PD on a previous AHS survey. Of the 345 self-reported cases, 183 (53%) had PD recorded as the underlying or contributing cause of death. Of the remaining 156 identified only from death certificates, 21% had participated in one follow-up survey, and 47% had participated in one or more. We obtained a PD screener for 232 deceased participants, including 47 from participants or their proxy while patients were alive and the rest from the proxy after their death. Of these 232, 47 had a death certificate as the only source of PD, 103 had both death certificate and self-report, and 82 only self-report (S4 Table). Of the 47 identified only from a death certificate, 57% met the diagnostic criteria for “probable” or “possible” PD based on proxy screener, although we note that we relaxed the diagnostic criteria for deceased with PD indicated on the death certificate (S1 Table). Of the 82 deceased with PD indicated only by self-report, 57% were classified as “probable” or “possible PD”.

## Discussion

In large prospective studies, due to practical constraints associated with physician-based standardized PD ascertainment, researchers may instead want to rely on self-report of doctor-diagnosis to identify PD cases. Few studies have explored how well self-reported diagnosis compares with clinical diagnostic criteria. In this cohort of farmers and spouses with over 20 years of follow-up, we found that 77.6% participants who self-reported physician diagnosis of PD were classified as “probable” or “possible” PD using Gelb’s diagnostic criteria [15]. Further, when we compared recently self-reported PD-related information in Phase 4 with the PD screener data, we found that participants who reported being diagnosed by a specialist, currently taking medications, or having multiple motor symptoms were more likely to be classified as “probable” or “possible” PD. Further, for a subset of 65 participants whose medical records were obtained, 93.4% of screener adjudicated PD were confirmed by medical record review.

We found that potential PD cases endorsing motor and non-motor symptoms in earlier AHS surveys had higher odds of being confirmed using the diagnostic criteria. These symptoms were ascertained as part of the cohort’s general follow-up, 2 to 10 years before the current PD screener data collection from potential PD patients; PD adjudication was entirely based on PD screener data and therefore independent of the cohort-wide motor and nonmotor data collection. Further, being a nonsmoker was strongly associated with “screener-confirmed PD”, supporting the validity of the approach we employed. Smoking has consistently been associated with reduced PD risk [18]. Both impaired olfaction and dream-enacting behavior, a characteristic feature of rapid-eye-movement sleep behavior disorder, are among the earliest manifestations of prodromal PD; impaired olfaction is considered the most sensitive and rapid-eye-movement sleep behavior disorder the most specific non-motor symptom of PD [17]. It is notable that self-reported motor and nonmotor symptoms can be readily collected as part of the health follow-up of large prospective cohort studies, presenting an opportunity to both help adjudicate PD diagnosis and to prospectively study the dynamic PD prodromal development longitudinally.

Use of multiple sources including self-reports, medication inventories, hospitalization data, and medical records for PD identification is common in epidemiologic studies [2–8]. A few studies have compared self-reported PD with clinical diagnostic criteria or medical records and reported good agreement. For example, a study using data from the Framingham Heart Study and the Framingham Offspring Study found high concordance between self-report (i.e., positive response to if they had ever been diagnosed with PD or whether they had been ever hospitalized because of PD) and clinical diagnostic criteria, with sensitivity of 93% and perfect specificity and positive predictive value, although high performance of self-report was attributed to higher education and greater health care access in this population [11]. Likewise, in the Women’s Health and Aging Study I, the agreement between self-report of physician diagnosis of PD and medical record was also very high (sensitivity: 89%; specificity, positive predictive value, and negative predictive value:100%) [9]. Another study, that employed in-person examination, medical records, or data from prior research to validate PD in participants (recruited based on self-report of physician diagnosis of PD) in the Washington State Parkinson Disease Registry, found that 93.4% of the registry participants met the UK Biobank criteria for PD [10]. Lastly, in the FAME study, 84% of self-reports were confirmed by clinical diagnostic criteria [14]. Another investigation based in the Netherlands found that 62% of PD self-report collected in study surveys were in agreement with general practitioner-validated cases, comparable to or higher than some of other sources including hospital discharge registry [6].

Our observation that only 53% of those who self-reported PD had PD recorded as the underlying or contributing cause of death was similar to other US-based reports of 54.8% [19] and 51.5% [20], and thus in line with existing literature suggesting underrepresentation of PD on death certificates. On the other hand, we identified 156 participants with PD recorded as a cause of death from the death certificates alone, likely reflecting loss-to-follow up, or under-reporting during interviews; we obtained PD screeners for 47 such cases from proxy informants and found that 57% (n = 27) could be classified as “probable” or “possible” PD. The lower concordance is likely due to limits of proxy data.

Our study has several limitations. We considered diagnoses to be confirmed if supported by evidence from medical record review, although clinical diagnosis may not always agree with autopsy findings [21–23]. Further, we were only able to obtain medical records for 65 participants. It is possible that participants with confirmed PD or their physicians selectively participated more as compared to those whose PD was less well-established, thus overestimating the positive predictive value of self-reports. With a 65% response to the screener, our results may have been biased due to selective non-response by participants or proxies. However, non-response was largely from the potential proxies of participants who died early in the study, and for whom death certificate was the only source of PD identification. While Gelb et al. formulated the diagnostic criteria for use in clinical settings [15], we applied those criteria to self-reported data and relaxed some criteria specifically when participants were deceased, resulting in potential disease misclassification. Misclassification may also have occurred from incomplete response or less reliable response from participants or their proxy. For example, self-reported PD unconfirmed by screener was also associated with some characteristics common in PD, although associations were not as strong as for screener-confirmed PD, possibly due to under-confirmation due to missing information. It is also possible that those with unconfirmed PD had other diagnoses with overlapping symptoms. Another limitation of our study is that screener questions to ascertain PD motor symptom were not specific enough to distinguish motor symptoms types or underlying pathology. For example, our question “do your arms or legs shake” does not distinguish resting tremor from intention tremor. Further, we did not consider muscular rigidity in our definition, and our questions on bradykinesia (one of the most important characteristics according to the UK Parkinson’s Disease Society Brain Bank Clinical Diagnostic Criteria of the four cardinal symptoms) were not specific enough to distinguish bradykinesia from muscular rigidity. Nevertheless, these participants had either reported a physician-made diagnosis of PD or had PD recorded on a death certificate. Thus a priori probability for them truly having PD is high when these symptoms were reported.

Lastly, due to cost constraints and logistical reasons in this large cohort, we did not collect information from participants who did not report having PD in surveys or from those without PD recorded on death certificates. This limited our ability to identify PD cases that that were lost to follow up, and did not allow us to estimate the sensitivity and specificity of our screener-based algorithm.

Overall, in our study, self-report of physician diagnosis of PD alone, while agreeing reasonably well with diagnostic criteria, did not perform as well, as reported in some prior studies [9–11]. Lower performance of PD self-report in our current effort than in FAME may be because FAME used in-person clinical assessment while we relied on self-reports of symptoms for diagnosis and included symptoms information provided by proxies which themselves could be subject to misclassification. Among those who completed screeners, we found that PD self-report along with information on motor symptoms and PD medication showed good agreement with classification using both screener-based diagnostic criteria and medical records. However, these results should be interpreted with caution as we were unable to make similar comparisons among individuals from whom screeners could not be obtained.

## Supporting information

S1 Fig Timeline of Agricultural Health Study surveys, FAME study, and PD confirmation effort, Iowa and North Carolina, 1993–2016. (DOCX)

S1 Table Algorithm based on the Gelb diagnostic criteria. (DOCX)

S2 Table Classification of FAME participants applying the Gelb criteria on self- or proxy- reported neurological symptoms and Parkinson’s Disease (PD) treatment reported at PD screener (n = 63) in the Agricultural Health Study, Iowa and North Carolina, 1993–2016. (DOCX)

S3 Table Motor and non-motor symptoms reported in Phase 3 and Phase 4 in relation to “screener-confirmed” and “unconfirmed” PD^a^ in the Agricultural Health Study, Iowa and North Carolina, 1993–2016. (DOCX)

S4 Table Comparison of Parkinson’s Disease (PD) classified by the Gelb criteria with death certificate information in the Agricultural Health Study, Iowa and North Carolina, 1993–2016. (DOCX)

S1 Document Agricultural health study Parkinson’s disease supplemental screener (Telephone screener questions). (DOCX)
