# Supplementary material for: Parkinson’s disease case ascertainment in a large prospective cohort
Source: PLoS One. 2021 May 19;16(5):e0251852. doi: 10.1371/journal.pone.0251852 (PMC8133399; doi:10.1371/journal.pone.0251852)

**S1 Fig. Timeline of Agricultural Health Study Surveys, FAME study, and Parkinson’s disease (PD) confirmation effort, Iowa and North Carolina, 1993-2016**


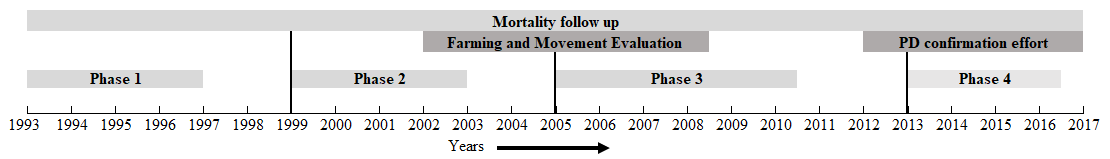

Supplement: S1 Fig — (DOCX) [file pone.0251852.s001.docx]
