# Supplementary material for: Parkinson’s disease case ascertainment in a large prospective cohort
Source: PLoS One. 2021 May 19;16(5):e0251852. doi: 10.1371/journal.pone.0251852 (PMC8133399; doi:10.1371/journal.pone.0251852)
Supplement: S1 Table — (DOCX) [file pone.0251852.s002.docx]

**S1 Table. Algorithm based on the Gelb diagnostic criteria**

| **Criteria** | **Gelb analogue for probable PD** | **AHS analogue** | **Definition** |
| --- | --- | --- | --- |
| 1 | Presence of all three features (resting tremor, bradykinesia, asymmetric onset) | Presence of all three features (resting tremor^a^, bradykinesia^b^, and asymmetric onset^c^) if participants interviewed;  Presence of 2 features (one must be resting tremor^a^ or bradykinesia^b^) if proxy interviewed | Probable PD if one of the following set of criteria was met   - [1,2,3,5] - [2,3,4] |
| 2 | Substantial and sustained response to therapy | For any medications (carbidopa, levodopa, pramipexole, ropinirole, pergolide, selegiline, rasagiline) –“yes” to taking for ≥ 1 year and still taking OR taken these medications ≥ 3 years even discontinued) |  |
| 3 | Disease duration ≥ 3 years | Disease duration ≥ 3 years |  |
| 4 |  | Diagnosed by movement disorder specialist (no Gelb analogue) |  |
| 5 |  | Diagnosed by neurologist (no Gelb analogue) |  |
|  | **Gelb analogue for possible PD** |  |  |
| 6 | Presence of two of the three features (one is resting tremor or bradykinesia) | If proxy interviewed, presence of one feature (but must be tremor^a^ or bradykinesia^b^) | Possible PD if one of the following set of criteria was met   - [6,2] - [5 AND (2 OR 9)] - [4 AND (8 OR 9)] - [7] - [If deceased, diagnosed by neurologist AND 13 and [10 OR 11 OR 12] AND no conflicting information] |
| 7 | If qualify for probable PD but duration < 3 years | Disease duration < 3 years |  |
| 8 | Substantial or sustained response to therapy OR no therapy | For any medications (carbidopa, levodopa, pramipexole, ropinirole, pergolide, selegiline, rasagiline) – “yes” to taking for ≥ 1 year and still taking OR taken these medications ≥ 3 years even discontinued OR never took any of the medications  If proxy interviewed and responded positively to the question “if medication helped”, then considered response to therapy regardless of years taken (may not be able to calculate) |  |
| 9 |  | Resting tremor^a^ (no Gelb analogue) |  |
| 10 |  | Resting tremor^a^ or bradykinesia^b^ (no Gelb analogue) |  |
| 11 |  | Ever took any medications (carbidopa, levodopa, pramipexole, ropinirole, pergolide, selegiline, rasagiline) (no Gelb analogue) |  |
| 12 |  | PD diagnosis by neurologist or movement disorder specialist (no Gelb analogue) |  |
| 13 |  | PD (ICD-9 332.0) on death certificate (no Gelb analogue) |  |

Note: ^a^Resting tremor: positive response to “do your arms or legs shake?”; ^b^Bradykinesia: positive response to any of the questions: do you have trouble rising from a chair, do your feet ever seem to get stuck to the floor, do you have trouble buttoning buttons, do you shuffle your feet and/or take tiny steps when you walk, do you move more slowly that other people your age, is your handwriting smaller than it once was, do people tell you that your voice is softer than it once was, and do people tell you that your face seems less expressive than it once did?; ^c^Asymmetric onset: positive response to any two question: did any of your symptoms start on only one side of your body; and were any of these symptoms more severe on one side of your body compared to the other side?

Abbreviations: AHS, Agricultural Health Study; PD, Parkinson’s Disease; ICD-9, International Classification of Diseases, 9^th^ Revision
