# Supplementary material for: Parkinson’s disease case ascertainment in a large prospective cohort
Source: PLoS One. 2021 May 19;16(5):e0251852. doi: 10.1371/journal.pone.0251852 (PMC8133399; doi:10.1371/journal.pone.0251852)
Supplement: S2 Table — (DOCX) [file pone.0251852.s003.docx]

**S2 Table: Classification of FAME Participants Applying the Gelb Criteria on Self- or Proxy- reported Neurological Symptoms and Parkinson's Disease (PD) Treatment Reported at PD Screener (n=63) in the Agricultural Health Study, Iowa and North Carolina, 1993-2016**

| PD Classification^a^ | Case (n=62) | |  | Control (n=1) | |
| --- | --- | --- | --- | --- | --- |
|  | n | % |  | n | % |
| Probable | 37 | 59.7 |  | 0 | 0 |
| Possible | 20 | 32.3 |  | 1 | 100 |
| Questionable | 2 | 3.2 |  | 0 | 0 |
| Other neurological condition | 2 | 3.2 |  | 0 | 0 |
| No PD | 1 | 1.6 |  | 0 | 0 |

^a^ Self-reported (or proxy-reported when participants were deceased or ill) information on the screener was evaluated using criteria analogous to the established diagnostic criteria to classify potential PD into “probable”, “possible”, “questionable”, “other neurological condition”, and “no PD”
