# Supplementary material for: Parkinson’s disease case ascertainment in a large prospective cohort
Source: PLoS One. 2021 May 19;16(5):e0251852. doi: 10.1371/journal.pone.0251852 (PMC8133399; doi:10.1371/journal.pone.0251852)
Supplement: S3 Table — (DOCX) [file pone.0251852.s004.docx]

**S3 Table: Motor and Non-motor Symptoms Reported in Phase 3 and Phase 4 in Relation to “Screener-confirmed” and “Unconfirmed” Parkinson’s Disease (PD)^a^ in the Agricultural Health Study, Iowa and North Carolina, 1993-2016**

| Parkinson’s disease symptoms | Non cases (n=83,879)^b^ | | Screener-confirmed PD^a^ (n=383)^b^ | | | | Unconfirmed PD^a^ (n=127)^b^ | | | |
| --- | --- | --- | --- | --- | --- | --- | --- | --- | --- | --- |
|  | N | % | n | % | OR^c^ | 95% CI | n | % | OR^c^ | 95% CI |
| **Motor symptoms in Phase 3 (during last 12)** |  |  |  |  |  |  |  |  |  |  |
| Hands shake? |  |  |  |  |  |  |  |  |  |  |
| No | 39692 | 92.5 | 130 | 48.3 | 1.0 |  | 66 | 71 | 1.0 |  |
| Yes | 3220 | 7.5 | 139 | 51.7 | 10.2 | 7.9, 13.1 | 27 | 29 | 3.7 | 2.3, 5.8 |
| Arms or legs shake? |  |  |  |  |  |  |  |  |  |  |
| No | 41439 | 96.6 | 160 | 59.5 | 1.0 |  | 72 | 77.4 | 1.0 |  |
| Yes | 1472 | 3.4 | 109 | 40.5 | 16.6 | 12.8, 21.6 | 21 | 22.6 | 6.6 | 4.0, 10.9 |
| Handwriting smaller than it once was? |  |  |  |  |  |  |  |  |  |  |
| No | 39527 | 93.2 | 130 | 49.4 | 1.0 |  | 69 | 77.5 | 1.0 |  |
| Yes | 2902 | 6.8 | 133 | 50.6 | 9.8 | 7.6, 12.6 | 20 | 22.5 | 2.4 | 1.4, 4.0 |
| People say your voice is softer than before? |  |  |  |  |  |  |  |  |  |  |
| No | 38668 | 91.9 | 142 | 55.3 | 1.0 |  | 60 | 65.2 | 1.0 |  |
| Yes | 3411 | 8.1 | 115 | 44.7 | 5.7 | 4.4, 7.3 | 32 | 34.8 | 3.6 | 2.3, 5.7 |
| Shuffle feet or take tiny steps? |  |  |  |  |  |  |  |  |  |  |
| No | 40607 | 94.9 | 165 | 61.3 | 1.0 |  | 66 | 71 | 1.0 |  |
| Yes | 2192 | 5.1 | 104 | 38.7 | 7.2 | 5.5, 9.3 | 27 | 29 | 4.5 | 2.8, 7.3 |
| Have a problem with your balance? |  |  |  |  |  |  |  |  |  |  |
| No | 37626 | 87.7 | 145 | 53.9 | 1.0 |  | 58 | 62.4 | 1.0 |  |
| Yes | 5264 | 12.3 | 124 | 46.1 | 4.3 | 3.3, 5.6 | 35 | 37.6 | 2.6 | 1.7, 4.1 |
| Move more slowly than others your age? |  |  |  |  |  |  |  |  |  |  |
| No | 38617 | 90.8 | 149 | 56.4 | 1.0 |  | 63 | 68.5 | 1.0 |  |
| Yes | 3913 | 9.2 | 115 | 43.6 | 6.3 | 4.9, 8.1 | 29 | 31.5 | 3.4 | 2.1, 5.3 |
| No. of motor symptoms |  |  |  |  |  |  |  |  |  |  |
| 0 to 2 | 40698 | 94.8 | 123 | 45.7 | 1.0 |  | 63 | 67.7 | 1.0 |  |
| 3 to 4 | 1797 | 4.2 | 59 | 21.9 | 7.9 | 5.7, 11.0 | 15 | 16.1 | 3.3 | 1.8, 5.9 |
| 5 to 7 | 431 | 1.0 | 87 | 32.3 | 52.2 | 38.2, 71.4 | 15 | 16.1 | 14.2 | 7.8, 25.7 |
| **Motor symptoms reported in Phase 4 (ever)** |  |  |  |  |  |  |  |  |  |  |
| Hands shake or tremble? |  |  |  |  |  |  |  |  |  |  |
| No | 35542 | 93 | 63 | 28 | 1.0 |  | 38 | 54.3 | 1.0 |  |
| Yes | 2678 | 7.0 | 162 | 72 | 29.2 | 21.7, 39.4 | 32 | 45.7 | 8.5 | 5.3, 13.7 |
| Arms or legs shake? |  |  |  |  |  |  |  |  |  |  |
| No | 37376 | 97.9 | 106 | 47.5 | 1.0 |  | 52 | 74.3 | 1.0 |  |
| Yes | 815 | 2.1 | 117 | 52.5 | 46.8 | 35.2, 62.2 | 18 | 25.7 | 11.6 | 6.7, 20.2 |
| Handwriting smaller? |  |  |  |  |  |  |  |  |  |  |
| No | 34795 | 91.5 | 42 | 18.8 | 1.0 |  | 38 | 55.1 | 1.0 |  |
| Yes | 3222 | 8.5 | 182 | 81.3 | 41.5 | 29.2, 59.1 | 31 | 44.9 | 5.3 | 3.2, 8.8 |
| Voice softer? |  |  |  |  |  |  |  |  |  |  |
| No | 34088 | 90.1 | 47 | 21 | 1.0 |  | 37 | 53.6 | 1.0 |  |
| Yes | 3766 | 9.9 | 177 | 79 | 27.2 | 19.4, 38.1 | 32 | 46.4 | 4.8 | 2.9, 7.9 |
| Feet shuffle when walking? |  |  |  |  |  |  |  |  |  |  |
| No | 35780 | 93.9 | 73 | 32.7 | 1.0 |  | 41 | 58.6 | 1.0 |  |
| Yes | 2306 | 6.1 | 150 | 67.3 | 24.8 | 18.3, 33.6 | 29 | 41.4 | 6.5 | 3.9, 10.8 |
| Trouble rising from chair? |  |  |  |  |  |  |  |  |  |  |
| No | 31017 | 81.4 | 58 | 25.7 | 1.0 |  | 36 | 52.2 | 1.0 |  |
| Yes | 7099 | 18.6 | 168 | 74.3 | 10.5 | 7.6, 14.4 | 33 | 47.8 | 2.4 | 1.5, 3.9 |
| No. of motor symptoms |  |  |  |  |  |  |  |  |  |  |
| 0 to 2 | 36308 | 94.8 | 36 | 15.9 | 1.0 |  | 35 | 50 | 1.0 |  |
| 3 to 4 | 1698 | 4.4 | 79 | 35 | 48.5 | 31.8, 74.0 | 22 | 31.4 | 8.2 | 4.6, 14.5 |
| 5 to 6 | 285 | 0.7 | 111 | 49.1 | 449.8 | 293, 690.6 | 13 | 18.6 | 28.9 | 14.6, 57.3 |
| **Non-motor symptoms reported in Phase 4** |  |  |  |  |  |  |  |  |  |  |
| Loss or decreased sense of smell? |  |  |  |  |  |  |  |  |  |  |
| No | 35073 | 91.9 | 101 | 45.3 | 1.0 |  | 51 | 71.8 | 1.0 |  |
| Yes | 3103 | 8.1 | 122 | 54.7 | 10.9 | 8.3, 14.3 | 20 | 28.2 | 3.3 | 2.0, 5.6 |
| Act out dreams? |  |  |  |  |  |  |  |  |  |  |
| No | 35721 | 93.5 | 139 | 61.5 | 1.0 |  | 62 | 87.3 | 1.0 |  |
| Yes | 2502 | 6.5 | 87 | 38.5 | 8.5 | 6.4, 11.2 | 9 | 12.7 | 2.0 | 1.0, 4.0 |

^a^ We categorized potential PD cases that met the criteria for “probable” or “possible” PD (when PD screener data were evaluated) as “screener-confirmed PD” and those that did not as “unconfirmed PD”, and the rest of the cohort with no evidence of PD as “non-cases”.

^b^Numbers do not add up to the total because of non-participation in Phase 3 and Phase 4

^c^Adjusted for age (continuous), sex, state, smoking, and education (all baseline variables); adjustment for Phase 3 or Phase 4 variables (when relevant) provides similar estimates % of self-reported PD confirmed by screener

Abbreviations: OR, Odds Ratio; CI, Confidence Interval
