# Supplementary material for: Parkinson’s disease case ascertainment in a large prospective cohort
Source: PLoS One. 2021 May 19;16(5):e0251852. doi: 10.1371/journal.pone.0251852 (PMC8133399; doi:10.1371/journal.pone.0251852)
Supplement: S4 Table — (DOCX) [file pone.0251852.s005.docx]

**S4 Table. Comparison of Parkinson's Disease (PD) Classified by the Gelb Criteria With Death Certificate Information in the Agricultural Health Study, Iowa and North Carolina, 1993-2016**

| PD Classification^a^ | PD source: death records only (n=47) | |  | PD Yes in death records & also self-reported (n=103) | |  | PD No in death records but self-reported (n=82) | |
| --- | --- | --- | --- | --- | --- | --- | --- | --- |
|  | n | % |  | n | % |  | n | % |
| Probable | 7 | 14.9 |  | 39 | 37.9 |  | 16 | 19.5 |
| Possible | 20 | 42.6 |  | 54 | 52.4 |  | 31 | 37.8 |
| Questionable | 1 | 2.1 |  | 2 | 1.9 |  | 6 | 7.3 |
| Other neurological condition | 7 | 14.9 |  | 6 | 5.8 |  | 13 | 15.9 |
| No PD | 12 | 25.5 |  | 2 | 1.9 |  | 16 | 19.5 |

^a^ Self-reported (or proxy-reported when participants were deceased or ill) information on the screener was evaluated using criteria analogous to the established diagnostic criteria to classify potential PD into “probable”, “possible”, “questionable”, “other neurological condition”, and “no PD”
