# Supplementary material for: Parkinson’s disease case ascertainment in a large prospective cohort
Source: PLoS One. 2021 May 19;16(5):e0251852. doi: 10.1371/journal.pone.0251852 (PMC8133399; doi:10.1371/journal.pone.0251852)
Supplement: S1 Document — (DOCX) [file pone.0251852.s006.docx]

**S1 Document. Agricultural Health Study Parkinson’s Disease Supplemental Screener (Telephone Screener Questions)**

1. Can you tell me if a doctor has ever diagnosed you with Parkinson’s disease?

[Yes, Probably Yes, No, Don’t know]

1. How old were you when a doctor first diagnosed you with Parkinson’s disease?
   1. Age [ ]
   2. What year were you first diagnosed with Parkinson’s disease? [ ]
   3. About how long ago were you first told you had Parkinson’s disease?
   4. [< 5 years ago, 5-10 years ago, > 10 years ago]
2. Has your diagnosis changed?

[Yes, No, Don’t Know]

1. Were you ever diagnosed with one of the following conditions? OR what is your current diagnosis?
   1. Essential tremor [Yes, No, Don’t know]
   2. Dementia with Lewy bodies or DLB [Yes, No, Don’t know]
   3. Progressive supranuclear palsy or PSP [Yes, No, Don’t know]
   4. Multiple system atrophy or MSA [Yes, No, Don’t know]
   5. Shy Drager syndrome [Yes, No, Don’t know]
   6. Nigrostriatal degeneration [Yes, No, Don’t know]
   7. Other neurologic disease (specify) [Yes, No, Don’t know]
2. Was your current diagnosis made or confirmed by a neurologist? [Yes, No, Don’t know]
3. Was the neurologist a movement disorders specialist? (A movement disorders specialist is a neurologist with special training in Parkinson’s disease and related conditions).

[Yes, No, Don’t know]

1. Please answer the following questions as they relate to your usual way of life in recent times.
   1. Do you have trouble rising from chair? [Yes, No, Uncertain] [Age/Year started]
   2. Is your balance poor? [Yes, No, Uncertain] [Age/Year started]
   3. Do your feet ever seem to get stuck to the floor? [Yes, No, Uncertain] [Age/Year started]
   4. Do your arms or legs shake? [Yes, No, Uncertain] [Age/Year started]
   5. Do you have trouble buttoning buttons? [Yes, No, Uncertain] [Age/Year started]
   6. Do you shuffle your feet and/or take tiny steps when you walk? [Yes, No, Uncertain] [Age/Year started]
   7. Do you move more slowly than other people your age? [Yes, No, Uncertain] [Age/ Year started]
   8. Is your handwriting smaller than it once was? [Yes, No, Uncertain] [Age/Year started]
   9. Do people tell that your voice softer than it once was? [Yes, No, Uncertain] [Age/ Year started]
   10. Do people tell you that your face seems less expressive than it once did? [Yes, No, Uncertain] [Age/ Year started]
2. Did any of your symptoms start on only one side of your body? [Yes, No, Don’t know]
3. Were any of these symptoms ever more severe on one side of your body compared to the other side? [Yes, No, Don’t know]
4. Did you ever take Carbidopa or levodopa such as Sinemet, Stalevo, or Parcopa for more than a month? [Yes, No, Don’t know]
   1. How old were you when you first started this medication?
      1. Age
      2. What year was it when you first started taking medication?
   2. Do you feel that it helped? [Yes, No, Don’t know]
   3. Are you still taking it? [Yes, No, Don’t know]
5. Did you ever take Mirapex or Pramipexole for more than a month?
   1. Similar questions as 10a through 10c
6. Did you ever take Requip or Ropinirole for more than a month?
   1. Similar questions as 10a through 10c
7. Did you ever take Permax or Pergolide for more than a month?
   1. Similar questions as 10a through 10c
8. Did you ever take Eldepryl or Selegiline for more than a month?
   1. Similar questions as 10a through 10c
9. Did you ever take Rasagline or Azilect for more than a month?
   1. Similar questions as 10a through 10c
10. Was anyone else in your immediate family ever told by a doctor that they had Parkinson’s disease? Your immediate family includes your biological (natural) parents, full (whole) brothers or sisters, and biological children. [Yes, No, Don’t Know]
11. Please tell me who in your family had a diagnosis of Parkinson’s disease?
    1. What was their age at diagnosis?
    2. Did the diagnosis change?
    3. What is (was) the current (final) diagnosis?
    4. What is their present age (or how old were they when died?)
12. Post interview ratings
    1. Please evaluate your confidence in the participant’s answers [Very confident/confident, Questionable, Unreliable]
    2. Did the participant have any difficulties in the interview? [Yes, No]
    3. Which difficulties did participant have? [Hearing, Cognitive (e.g., memory), Speaking, Other (specify)]
    4. Who responded to the questions regarding this participant? [Participant, Participant with help]
    5. Other comments?
